# Do Informational and Emotional Elements Differ between Online Psychological and Physiological Disease Communities in China? A Comparative Study of Depression and Diabetes

**DOI:** 10.3390/ijerph19042167

**Published:** 2022-02-15

**Authors:** Zhizhen Yao, Zhenni Ni, Bin Zhang, Jian Du

**Affiliations:** 1 School of Information Management, Wuhan University, Wuhan 430072, China; yaozhizhen@whu.edu.cn (Z.Y.); jennie_n@whu.edu.cn (Z.N.); 2 Center for the Studies of Information Resources, Wuhan University, Wuhan 430072, China; 3 Department of Information Systems, College of Business, City University of Hong Kong, Hong Kong 999077, China; 4 School of Information Management, Nanjing University, Nanjing 210023, China; 5 National Institute of Health Data Science, Peking University, Beijing 100191, China; dujian@bjmu.edu.cn

**Keywords:** online disease community, emotional contagion, topic mining, sentiment polarity, social network analysis, chronic disease

## Abstract

Disease-specific online health communities provide a convenient and common platform for patients to share experiences, change information, provide and receive social support. This study aimed to compare differences between online psychological and physiological disease communities in topics, sentiment, participation, and emotional contagion patterns using multiple methods as well as to discuss how to satisfy the users’ different informational and emotional needs. We chose the online depression and diabetes communities on the Baidu Tieba platform as the data source. Topic modeling and theme coding were employed to analyze discussion preferences for various topic categories. Sentiment analysis was used to identify the sentiment polarity of each post and comment. The social network was used to represent the users’ interaction and emotional flows to discover the differences in participation and emotional contagion patterns between psychological and physiological disease communities. The results revealed that people affected by depression focused more on their symptoms and social relationships, while people affected by diabetes were more likely to discuss treatment and self-management behavior. In the depression community, there were obvious interveners spreading positive emotions and more core users in the negative emotional contagion network. In the diabetes community, emotional contagion was less prevalent and core users in positive and negative emotional contagion networks were basically the same. The study reveals insights into the differences between online psychological and physiological disease communities, providing a greater understanding of the users’ informational and emotional needs expressed online. These results are helpful for society to provide actual medical assistance and deploy health interventions based on disease types.

## 1. Introduction

### 1.1. Background

The World Health Organization [1] reported that “chronic diseases are the leading causes of death worldwide and the disease rates from these conditions are accelerating globally, advancing across every region and pervading all socioeconomic classes”. Compared with other diseases, people suffering from chronic diseases often require a long period of treatment, and the disease becomes a part of their daily life. Therefore, they have many special informational needs and emotional needs around the disease over a long period of time. With the popularization of the Internet and the strengthening of public health awareness, patients with chronic diseases are more willing to seek informational and emotional support online. Online health communities (OHCs) play a strong role in providing support, where people with health problems can share objective and experiential information with others and seek sensitive topics via anonymity [2]. User-generated content (UGC) online is becoming a valuable resource for researchers to study patients’ needs and behaviors [3]. Topic modeling [4], sentiment analysis [5], and social network analysis [6] are often used to analyze UGC to uncover topics, emotions, and behaviors hidden in online communities.

Mental health and physical health are two important health issues in chronic diseases that are receiving more and more attention. Dividing communities according to the disease type, and then studying internal informational and emotional elements, is conducive to deepening the understanding of the online behavior of patients with different diseases. On the basis of these discovered differences, personalized interventions and support behaviors can be developed [7,8]. Chronic diseases can be divided into physiological diseases and psychological diseases. Chronic mental disorders mainly include depression, personality disorders, etc. Chronic physical diseases mainly include diabetes, stroke, cardiovascular disease, etc. As both of these types of diseases are chronic, patients with physiological and psychological diseases share some similarities in terms of discussion topics, emotional expression, and engagement patterns, but there are also differences due to different disease types.

### 1.2. Online Disease Communities

One of the leading sources of health information online is from online health communities. Users of OHCs can acquire knowledge and advice from others as well as share objective and experiential information [9]. Online disease communities (ODCs) link people who have common diseases with each other. Compared with OHCs, the topics discussed by users in ODCs are more closely related to a specific disease. Even with strong networks of support from family and friends, ODCs are valuable and important resources that make it possible for individuals to interact with patients with the same disease and obtain social support from them [10]. A wide range of ODC studies have been carried out to study health topics and user needs [4], create social support interventions [11], and understand patient sentiment [12] and behaviors [13].

Previous studies on ODCs have mostly focused on a single disease community such as cancer [14], diabetes [11], autism [15], and so on. There are few comparative studies on diseases in different communities. Some comparative studies of communities only focused on topics and emotions, and there was no further discussion on user participation behavior and different emotional contagion patterns. Regarding topics, Park et al. [7] found that the anxiety, depression, and PTSD communities shared four themes. Chen [16] compared breast cancer, type 1 diabetes, and fibromyalgia communities and found that the theme clusters fell into a set of common categories. Low et al. [17] compared fifteen mental health support groups on Reddit and found that “health anxiety emerged as a general theme across Reddit”. For emotions, Gkotsis et al. [18] found that negative sentiment was prevalent in the sixteen mental health communities. Patients with mental illness generally expressed more negative emotions online than those with physical illness [19]. Therefore, in order to better understand the characteristics of different disease communities and user groups, we divided the ODCs into psychological disease communities and physiological disease communities to carry out a comparative study.

### 1.3. Emotional Contagion Theory

Emotional contagion theory was defined as “a type of emotional influence that describes the spread of one person’s emotion to others during social encounters” [20], which plays a significant role in group members. It is widely used to identify opinion leaders [21,22] and improve the sentiment analysis model [23,24]. A study in PNAS collected over three million posts on Facebook from 698,003 users over a 20-year period and found that emotion could be transferred to others through emotional contagion, causing people to experience the same emotions unconsciously [25]. These positive and negative emotional states were proven to “behave like infectious diseases spreading across social networks over a long period of time” [26]. Negative emotional contagion, that is, catching someone else’s bad mood and experiencing an increase in negative mood as a result, is particularly common in ODCs. Among social networks, unpleasant emotions have been shown to be more likely to transfer to others than pleasant emotions [27]. At the same time, positive emotional support that has proven particularly beneficial to patients’ mental health can also be observed in ODCs [28]. Similarly, other desirable health outcomes can also be achieved through ODCs [29] including coping strategies [30], personal relationships [31], and even physical health [32]. In conclusion, figuring out the emotional contagion patterns of ODCs plays a significant role in patients’ physical and mental outcomes.

### 1.4. Objectives

The purpose of this study was to compare differences between psychological and physiological disease communities in informational and emotional elements including topic, sentiment, participation, and emotional contagion patterns using multiple methods. Based on these, personalized interventions and support can be provided to satisfy patients’ different informational and emotional needs due to different disease types. Specifically, this study aims to answer the following three questions (RQ).

RQ1: What are the main themes and how much thematic similarity and difference exists among the psychological and physiological disease communities?

RQ2: What are important features of sentiment and participation in psychological and physiological disease communities?

RQ3: What are the similarities and differences in emotional contagion patterns between psychological and physiological disease communities?

Depression and diabetes are two typical mental and physical disorders requiring a continuing network of support. Under these conditions, ODCs have unique value as communication platforms for people who share struggles with psychological or physiological diseases. Compared with previous studies, we innovatively introduce emotional contagion theory to understand positive and negative emotional contagion patterns of the two disease communities. We expect that quantifying differences between online psychological and physiological disease communities will yield valuable insights into specific informational and emotional elements expressed by patients with different types of disease and help deploy treatment more effectively.

## 2. Methods

**Figure 1:**
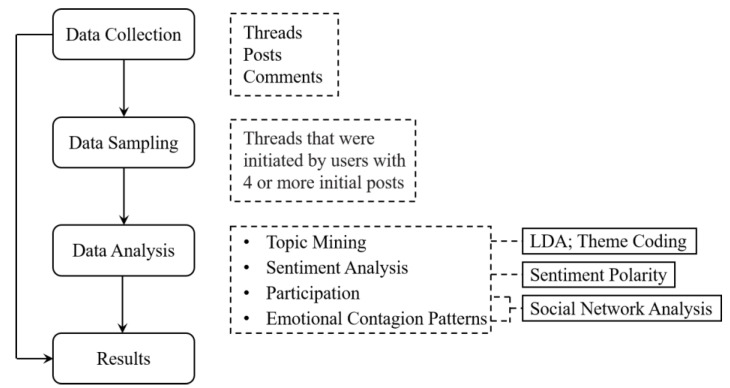
Research process to evaluate RQ1, RQ2, and RQ3.

Our approach employs topic modeling, theme coding, sentiment polarity analysis, and social network analysis to compare ODCs focusing on depression and diabetes. By comparing the themes and sentiment of UGC in the two communities as well as user engagement and emotional contagion patterns within the communities, the similarities and differences of the two ODCs in the above aspects can be obtained. The research process is described in Figure 1.

### 2.1. Data Collection

**Figure 2:**
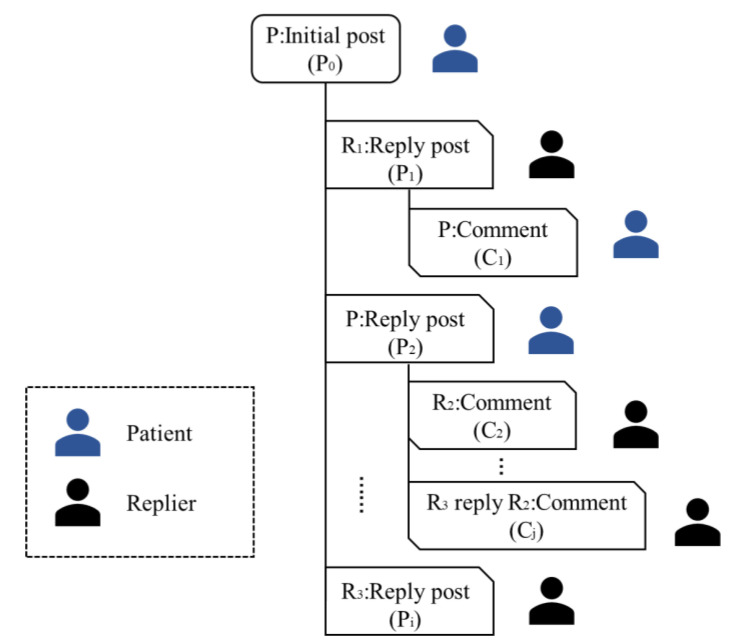
An example of a thread with posts and comments.

The dataset used in this study was collected from two online disease communities on Baidu Tieba, which is the largest Chinese online platform for self-disclosure and Q&A [33]. There are millions of communities providing places online where users can communicate with others, covering topics related to health, economy, entertainment, and so on [34]. To collect activity data on the users in online physiological and psychological disease communities, we chose the largest communities related to depression and diabetes on Baidu Tieba. The “depression community” and “diabetes community” had each respectively attracted 429,500 users and 186,006 users to contribute. There had been 1,583,885 threads in the depression community and 31,648 threads in the diabetes community by 1 April 2021. Figure 2 shows an example of a completed thread. Each threaded discussion begins with an initial post (P0) from a patient with depression or diabetes. This may be followed by several reply posts (P1, P2, …, Pi) including the self-replies (P2). To communicate with the repliers, the patient or someone else can reply to the posts by publishing comments (C1, C2). In addition, members can also reply to the comments under a post (Cj).

Accordingly, we designed a web spider using Python 3.7 to crawl the records dating from 1 January 2010 to 30 October 2020. Since some data collected before 2018 were severely lost, the dataset from 2018 to 2020 was eventually selected for subsequent analysis. During this time, we archived 12,040 threads including a total of 152,287 posts and 164,100 comments in the depression community and 8091 threads including a total of 65,688 posts and 76,218 comments in the diabetes community. After identifying and sampling the threads, we extracted the dataset that contained several fields, as shown in Table 1.

### 2.2. Data Sampling

Baidu Tieba is a public platform, where users can post without verifying their identities. Consequently, there are some advertisements and meaningless texts in the communities. Given the noise in the whole dataset, we restricted our analysis to threads that were initiated by users with four or more initial posts. This threshold ensured sufficient disease-related texts for analyses and was also used to determine that contributors who regularly expressed their views or emotions in ODCs are patients with depression or diabetes [35,36]. In addition, we removed posts and comments that were blank or had authors we could not recognize. Since users can modify their names, we used portrait, the suffix of the user’s homepage URL, as the unique identifier of the user.

The number of posts and comments generated by each user varied considerably, most users created only one thread, while few users actively generated the majority of posts and comments. A total of 6965 unique users initiated at least one thread in the depression community, 5418 of them published only one initial post, 465 users created a thread more than four times. They initiated a total of 4033 threads including 29,128 posts and 29,175 comments. The sample threads we extracted involved a total of 7100 users. In the diabetes community, 4147 unique users initiated at least one thread, 3088 of them published only one initial post, and 319 users who initiated a thread more than four times were identified. They initiated 3326 threads in total including 18,834 posts and 19,599 comments. A total of 3233 users were involved. Table 2 presents the basic descriptions of the collected dataset and sample dataset.

### 2.3. Topic Mining

To distinguish the differences in user discussion topics between physiological and psychological disease communities, we used the topic-generated model of LDA [37] to model the text of patient discussion. Latent Dirichlet Allocation (LDA) is a three-tier Bayesian topic-generation model, which assumes that each document has a different probability for each discussed topic. To ensure the amount and accuracy of topic mining, we treated a thread as a document for analysis, which contains the initial post and self-replies in the thread [38]. Conducting analysis at the thread-level ensures that all the data were indeed the sincere information needs expressed by patients. After identifying data for topic mining, we removed punctuation marks, URLs, and emoticons that were not related to the topic of the text. Then, Jieba 0.39 in Python 3.7 was employed for word segmentation. During the segmentation, we used the Chinese Medical Subject Headings to expand the lexical dictionary due to the particularity of diseases and removed stop words by the stop word list of the Harbin Institute of Technology in China. Finally, the words “depression” and “diabetes” were also removed to avoid having many topic words directly associated with the diseases. After word segmentation, threads that contained at least three words were preserved. The final dataset for topic mining contained 6930 records including 3806 depression threads and 3124 diabetes threads. 

Previous studies have shed light on the discussion topics in different ODCs. Based on the topic classification results of ODCs in the existing literature and the characteristics of our dataset, we constructed a generic theme classification scheme for ODCs. Each document has different probabilities of LDA categories. High probability words in some LDA categories may express the same theme. The core theme of LDA categories can be extracted and labeled manually corresponding to the theme classification scheme of ODCs to obtain the distribution probability of each document under each theme. Then, we conducted Mann–Whitney U tests to examine whether the differences in these theme arrays between the depression and diabetes communities were statistically significant.

### 2.4. Sentiment Polarity Identification

In order to find out the whole sentiment polarity of these two ODCs and the differences between initial posts, reply posts, and comments, TextMind [39] was applied to the text. TextMind is a Chinese language psychological analysis system developed by the Chinese Academy of Sciences. It provides easy access to analyze the preferences and degrees of different categories in text and returns each linguistic dimension score as a proportion of the total number of words under analysis.

The two most notable linguistic dimensions related to sentiment are “positive emotion” and “negative emotion”, both of which have been used in several studies to measure positive and negative emotion in the online users’ posts [40,41]. In this paper, sentiment polarity included three types: positive, neutral, and negative. We defined the sentiment polarity of our records as follows [42]: (1) If the score of positive emotion was higher than that of negative emotion, we defined the document as positive. On the contrary, we defined it as negative; and (2) if the score of positive emotion was equal to negative emotion, we defined it as neutral. All the records in the sample dataset were used for sentiment analysis.

### 2.5. Social Network Analysis

**Figure 3:**
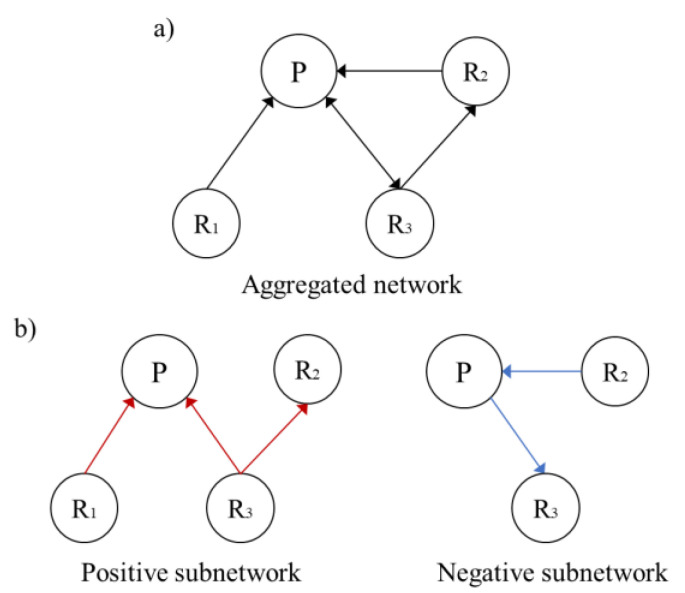
Corresponding networks including (**a**) an aggregated network and (**b**) positive and negative emotional contagion networks of the example in Figure 1.

Social network analysis has been widely used in studies of user interaction behavior [43] and social support [44,45] in online communities. In this study, social network analysis was conducted to represent emotional contagion and examine the basic social characteristics of different emotional contagion networks and the role of patients in emotional contagion. A node is defined as a user who posted at least a message in communities. A tie is operationalized as the post-reply relationship, that is, when A replied to B, a tie was established between them and the arrow pointed from A to B. We inferred that the sentiment of a user’s reply post is an objective manifestation of the user’s subjective desire to cause an emotional impact on other users. According to the sentiment polarity of each reply, the network can be divided into a positive emotional contagion network and a negative emotional contagion network. The aggregated network and subnetworks of the thread (Figure 1) are shown in Figure 3.

Ucinet 6.0 was employed in this study to construct two aggregated networks of ODCs and calculate the degree centrality of each node. Degree centrality is the number of direct relationships of an entity. A node with high degree centrality is generally an active user in the network. In directed networks, in-degrees of nodes reflect the number of replies one received, while the out-degrees indicate the replies one provided to others. After separating different sentiment polarities of posts and comments in ODCs, Gephi 0.9.2 was used to calculate and visualize the positive and negative emotional contagion networks.

## 3. Results

### 3.1. Preference Topics

**Figure 4:**
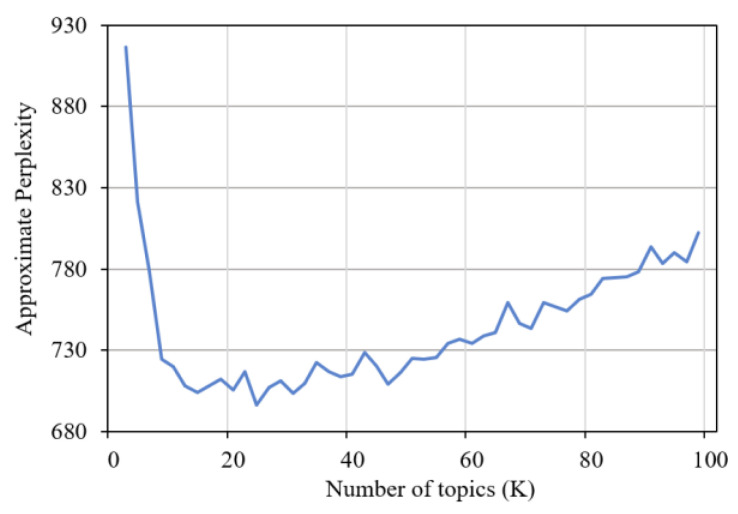
Latent Dirichlet Allocation model perplexity change graph.

Topic-generated model of LDA was used to identify the users’ preference topics in ODCs. To determine the optimal number of topics, we calculated the model perplexity in each number of topics (K). Based on the perplexity principle, a smaller value of perplexity reflects the greater clustering effect of the model [37]. As shown in Figure 4, the lowest perplexity was 24 (from 1 to 100). Therefore, we set the number of topics as 24 and generated the topic distribution for each document.

Moreover, after referring to previous literature and merging topics, a generic theme classification scheme for ODCs was formed, with a total of four main topics and eight subtopics, as shown in Table 3. In order to test the final classification scheme, an intercoder reliability test was conducted. Two students majoring in information science and medicine annotated each LDA category with the most related theme label. After labeling, Cohen’s Kappa coefficient was used to verify the consistency of the labeling results. The verification results showed that the Cohen’s Kappa coefficient was 0.855, indicating that the consistency results were good, so the classification scheme was reliable [46]. The coders again discussed and finally formed a consistent content annotation result. After LDA topic modeling, the topic probability distribution for each document could be obtained. Based on the annotation result, we measured the topical coverage of each subtopic by the sum of the topic probabilities of different LDA categories with the same label. For the concrete content of these 24 LDA categories and merging results, see Appendix A, Table A1.

**Figure 5:**
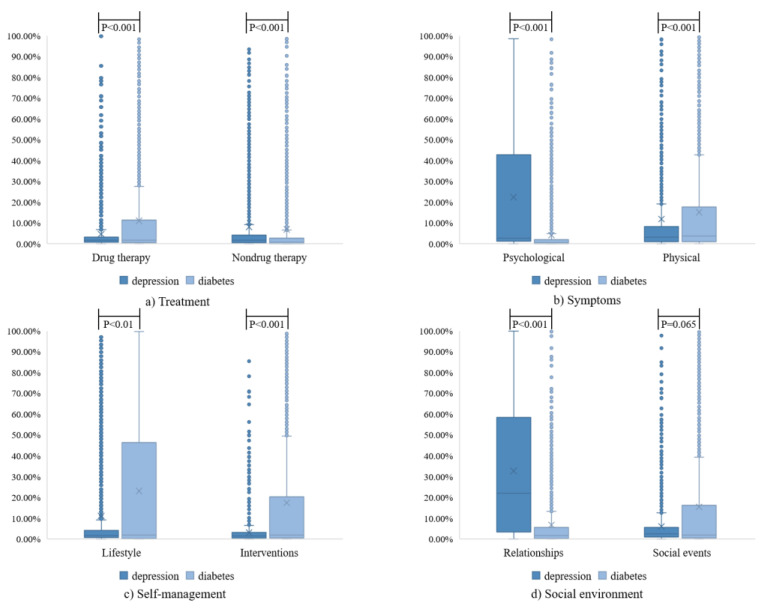
Document topic probability distribution of depression and diabetes communities. (**a**) The topic “Treatment”; (**b**) The topic “Symptoms”; (**c**) The topic “Experience”; (**d**) The topic “Social Environment”.

To assess the users’ discussion preferences and thematic differences in two disease communities, Figure 5 is displayed to analyze the document probability distribution of each topic in the communities. Nine discussed topics were found to have significant differences between the online depression and diabetes communities: drug therapy, nondrug therapy, psychological, physical, lifestyle, interventions, and relationships.

Figure 5 can clearly clarify the differences in the discussion preferences between the two ODCs. The first theme is treatment, which can be divided into drug therapy and nondrug therapy. Figure 5a shows that diabetics were more willing to discuss their medication habits in the community, while other treatment methods such as hospitalization and surgery were less likely to be discussed. Patients with depression paid less attention to treatment. The second theme is symptoms, which contains psychological and physical symptoms. It can be clearly observed in Figure 5b that patients with depression generally expressed a higher desire to talk about their mental states, and at the same time, they were also concerned about their physical states. Diabetics paid more attention to their physical symptoms and their mental states were not significantly affected by the disease. The third theme is experience and includes texts on lifestyle and interventions. As shown in Figure 5c, the online disease community was an important platform for diabetics to record their living habits and daily self-management behavior. Through this platform, they can restrain their behaviors, encourage, and supervise each other to take better care of themselves. In contrast, patients with depression did not pay much attention to their lifestyle and daily interventions for disease. The final theme is social environment, which includes texts on relationships and social events. It can be clearly seen in Figure 5d that patients with depression were more likely to talk about their relationships with others including parents, friends, work partners, etc. In contrast, diabetics worried less about their social relationships and more discussed hot spots of society.

### 3.2. Sentiment Polarity and Participation

#### 3.2.1. Sentiment Polarity of the Communities

**Figure 6:**
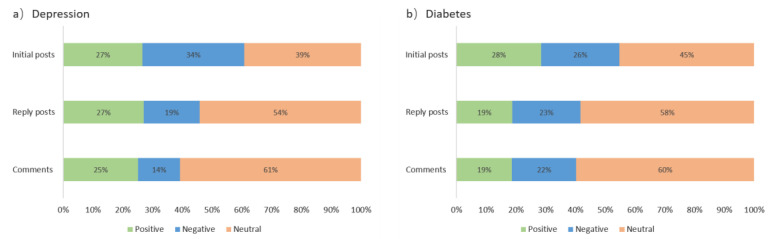
Proportion of text sentiment in online (**a**) depression and (**b**) diabetes communities.

Sentiment polarity of each initial post, reply post, and comment was identified and calculated. Figure 6 shows the proportion of text sentiment in the two communities after classification. In general, initial posts always contained more emotions, followed by reply posts and comments in ODCs. For positive emotion, it was distributed evenly among the three types of texts in the depression community, while in the diabetes community, it accounted for a large proportion in the initial posts and a small proportion in the reply posts and comments. For negative emotion, it accounted for a large proportion in the initial posts and gradually decreased in the reply posts and comments in the depression community, while the negative emotion of the diabetes community was evenly distributed in the three types of text. Compared with diabetics, patients with depression were more likely to initiate a thread to express their negative emotions, and there were more positive voices in the replies. Diabetics tended to post more positive content in their initial posts than those with depression, and users expressed less positive emotion in the response text.

**Figure 7:**
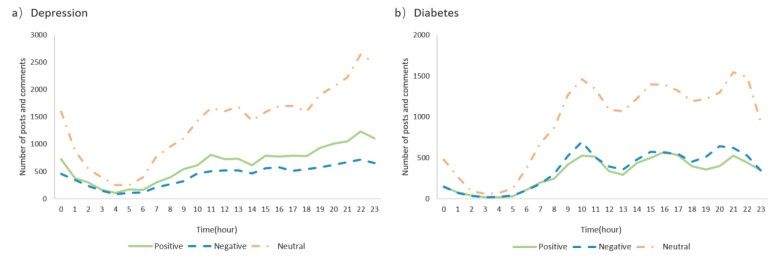
Distribution of positive, negative, and neutral texts by hour of the day in online (**a**) depression and (**b**) diabetes communities.

Figure 7 shows the number of posts and comments in different time periods, which reflects the users’ participation behavior. The line chart can also reflect the change of positive emotion, negative emotion, and neutral emotion over time in ODCs. The findings revealed that the users preferred to participate in the depression community after 18:00 p.m. and there was a peak at 22:00 p.m. Compared with positive and neutral emotions, negative emotion was stable and changed more slowly. In the diabetes community, three peak periods of use were clearly observed: the first was from 9:00 a.m. to 11:00 a.m., the second was from 15:00 p.m. to 17:00 p.m., and the third was from 20:00 p.m. to 22:00 p.m. The fluctuation range of positive emotion and negative emotion was basically the same. The peak of positive emotion in the afternoon and evening was one hour later than that of negative emotion.

#### 3.2.2. Post-Reply Relationships

**Figure 8:**
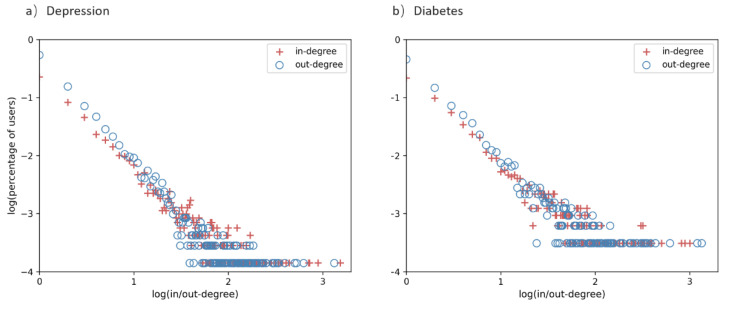
Distribution of users’ in/out-degree for the aggregated networks in online (**a**) depression and (**b**) diabetes communities.

Ucinet 6.0 was used to construct aggregated networks of two ODCs and calculate each user’s in-degree and out-degree. In aggregated networks, the higher in-degree of a member, the more replies the member received. The higher out-degree of a member, the more replies the member provided. There are 7100 nodes in the depression network and 3233 nodes in the diabetes network. In a log–log plot, the distributions of in-degree and out-degree in two aggregated networks approximate the long-tailed power-law (see Figure 8), which are typical for scale-free networks. This means that the majority of users have low in/out degrees while only a small proportion of users have very high in/out degrees in these two communities. Most posts were published by a few users and only a small number of users could receive the others’ replies.

### 3.3. Emotional Contagion Patterns

#### 3.3.1. Emotional Contagion Networks

The social network can portray the emotional contagion pattern among users. After identifying the sentiment polarity of each reply, the whole network can be divided into two kinds of emotional contagion networks: positive emotion and negative emotion. Table 4 shows the network measures calculated for each of four emotional contagion subnetworks. We compared the subnetworks with average degree, average clustering coefficient, and average path length. The average degree represents the average degree of nodes. The average clustering coefficient representing the degree of node aggregation in a graph. The small value of the clustering coefficient suggests that peer neighbors are not closely connected. The average path length is the average shortest distance between all pairs of nodes in the network. Suppose emotion is spread over a social network. The average clustering coefficient and path length measure the steps from A to B.

Different networks have different characteristics. Among the four subnetworks, the positive emotional contagion network of the depression community had the most nodes (3834), followed by the positive subnetwork of the diabetes community (3157). Overall, the nodes in the positive emotional contagion networks were more than those in the negative emotional contagion networks, indicating that more users participated in positive emotional contagion networks in ODCs. The number of ties and average degree of positive emotional contagion networks were higher than those of the negative emotional contagion networks, meaning that each person had fewer negative emotional connections and more positive emotional connections with others in both communities. In the depression community, the average distance between nodes was around 3.872 and the average clustering coefficient was around 0.014 for positive emotional contagion, which indicates that with fewer steps, positive emotion could reach other nodes than negative emotion. In contrast, the positive emotional contagion network had a higher average path length (3.550) and a lower average clustering coefficient (0.029) than the negative emotional contagion network in the diabetes community, which indicates negative emotion could spread more easily than positive emotion. In general, compared with the depression community, positive and negative subnetworks of the diabetes community had higher clustering coefficient and shorter average path length, though there were fewer nodes and ties in it. This means that information can spread among users more quickly in the diabetes community.

**Figure 9:**
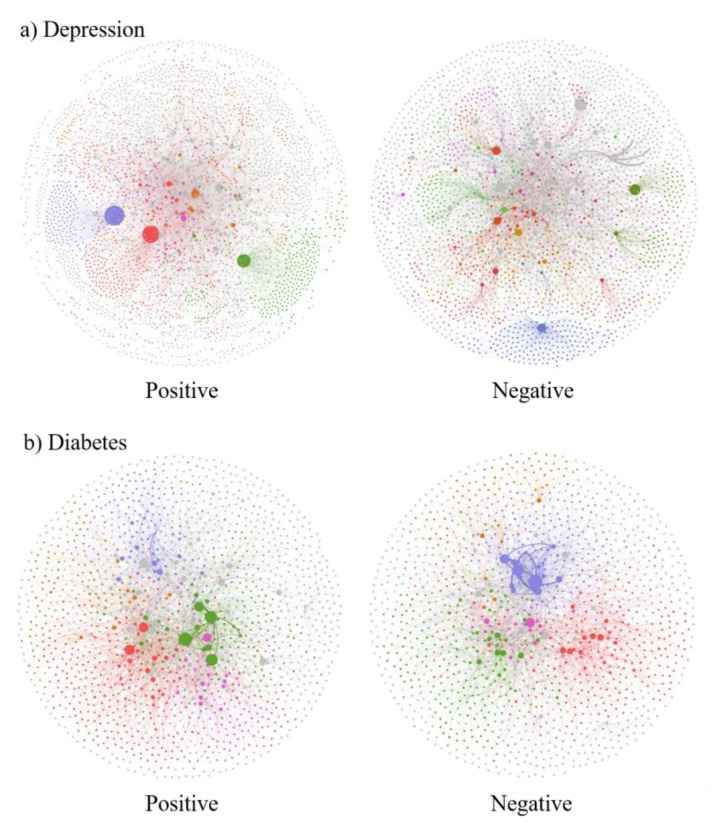
Positive and negative emotional contagion networks in online (**a**) depression and (**b**) diabetes communities.

Figure 9 presents the structure of different emotional contagion networks in ODCs. In these subnetworks, the node represents the user, and the node size is proportional to its out-degree, indicating the emotional intensity expressed by the user. The edge represents the strength of the emotional link between users, and the thicker the edge, the greater the amount of emotional contagion between users. The subnetworks can better capture how emotion flows among ODC users. The community detection algorithm is used to divide users, and nodes with the same characteristics are gathered together. Different communities are distinguished by color. The Louvain algorithm [53] was used to detect the potential community of participants and is recognized as one of the best methods for community detection in terms of computational time [54]. With such visualization, it is possible to see how different emotions can spread from core users to others and how they are positioned in the network.

It can be clearly observed that emotional contagion is more prevalent in the depression community than in the diabetes community. For the depression community, there were fewer core users with large emotional contagion volume in the positive emotional contagion network. The network structure was mainly manifested as the core users mainly expressing positive emotions and influencing lots of surrounding users. Finally, three obvious clusters were formed around core users, presenting an apparent core user-influenced emotional contagion dynamic. Compared with the positive emotional contagion network, the number of core users of the negative emotional contagion network was significantly higher, but the nodes were generally small. This means that more core users spread negative emotions in the network, but each user delivered a small amount of negative emotion. For the diabetes community, there were several core users in the positive and negative networks. The core nodes in these two networks were interconnected and emotions spread within different communities between users. This means that users in the diabetes community expressed and communicated their feelings with each other more frequently and there were no more influential users to spread emotions.

#### 3.3.2. Core Users and Interveners Identification

To explore differences between the core users, Table 5 presents the 10 participants who had the highest out degrees in the four subnetworks for the depression and diabetes communities. Descriptive network characteristics of these participants in four subnetworks are available in Appendix A, Table A2. For confidentiality reasons, the participants’ portraits were substituted with unique identifiers.

By comparing the data presented in Table 5, it is possible to infer that users who spread positive emotions are not completely different from those who spread negative emotions as only a few participants appeared in both positive and negative networks in the depression community. In contrast, seven of the top ten core users in the positive emotional contagion network could also be observed in the user list of the negative emotional contagion network in the diabetes community. This means that these participants spread negative emotions as well as positive emotions. Based on this, we defined the unique influential users in the positive emotional contagion network as “interveners”. Combined with Figure 9, it can be seen clearly that two obvious interveners (Dep_user_01, Dep_user_02) existed in the positive emotional contagion network of the depression community. They transmitted large positive emotions and had a huge impact on others around them, but rarely conveyed negative emotions. The core user Dep_user_03 was ranked high in both positive and negative subnetworks, so it cannot be defined as an intervener.

## 4. Discussion

### 4.1. Principal Findings

The study presents several significant findings about informational and emotional elements in online disease communities. We not only wanted to compare the overall discussion themes and the sentiment expressed by users in online depression and diabetes communities, but to also identify differences in emotional contagion patterns. Emotional contagion theory enables a better understanding of emotional contagion patterns of ODCs. As we expected, the depression and diabetes communities have some similarities and differences in the above aspects, and our findings well answer the three research questions raised at the beginning of this paper.

Preference topics: The discussion in the ODCs mainly focused on four topics: treatment, symptoms, self-management, and social environment. Patients with depression were more concerned about their own symptoms and social relationships with others. They were more likely to reveal their mental status and share their emotions in the community. Diabetics were more willing to share their life experiences within the community, and they tended to focus on their lifestyle and interventions in daily life. In sum, patients with mental illness paid more attention to the past, their own thoughts, and relationships with others, while patients with physiological disease had a more positive attitude toward the future and hoped to maintain fitness through treatment and self-management. 

Emotional expression: Patients were both willing to initiate a thread to express their negative emotions. As time passed, the number of posts in the depression community increased gradually from 6:00 in the morning and reached a peak at 22:00 in the evening. The online diabetes community had three emotional peaks during one day. Accordingly, compared with patients with physiological disease, patients with mental illness preferred to express their emotions at night.

Emotional contagion patterns: Core users of the positive emotional contagion network were fewer and different to those of the negative emotional contagion network in the depression community. This may suggest that positive emotions are conveyed more by the interveners than the patients in the depression community. In general, there were two obvious interveners in the depression community who broadly connected with other users and conveyed positive emotions to them. At the same time, positive emotions were more likely to spread within the community than negative emotion, so it is very important to bring in interveners and intervene proactively in the depression community. In contrast, core users in the diabetes community who spread positive and negative emotions were basically the same, and negative emotions spread more easily. Emotional contagion was less prevalent in the diabetes community. Therefore, more attention should be paid to the filtering of negative texts in the diabetes community.

Self-stigma: This difference in emotional contagion patterns provokes interesting discussions. One alternative explanation of the emotional contagion results could be self-stigma of depressed patients. People with mental disorders are often disdained by the public [55]. While all illnesses can face stigmatized attitudes from others, the public seems to discriminate against people with mental illnesses far more than those with physical illnesses [56]. “Stigmatized persons may internalize perceived prejudices and develop negative feelings about themselves”, the result of which is “self-stigma” [57]. Self-stigma has become an important barrier to expressing emotions and seeking treatment for depression and other mental illnesses [58,59]. Compared with physical diseases, the self-stigma of patients with mental illness makes them unwilling to accept help from others and afraid of discrimination. Such a negative view focuses too much on self and is difficult to resonate within the self-stigma group, thus hindering the spread of negative emotions in the community to some extent.

### 4.2. Implications

This study is significant in that it deepens the understanding of physiological and psychological diseases including the patients’ needs expressed on the Internet, their attitudes toward the disease, and the way of participating in online communities. On a theoretical level, we utilized emotional contagion theory to develop the study, and this research has helped to advance the understanding of how emotion flows in online communities and how they differ across different types of ODCs. On a practical level, for health care administrators and health care providers, our research can help provide corresponding social support according to their different information needs and emotional needs. Meanwhile, understanding the contagion patterns of different emotions in ODCs can provide insights for online community managers with suggestions on how to improve the patients’ mental well-being through interventions in different communities. Furthermore, this study addresses the psychosocial benefits of ODCs, and we should strengthen psychological counseling via ODCs. The ultimate goal of this research is to provide targeted social support and effective strategies for those who are struggling with psychological and physiological diseases, and contribute to improving their quality of life by making full use of ODCs. Meanwhile, ODCs should consider user privacy when deploying any intervention.

### 4.3. Limitations and Future Directions

There are some limitations that may encourage further research efforts. First, because of the particularity of each community, the users’ needs, emotions, and engagement patterns may differ. Although depression and diabetes are two typical chronic psychological and physiological diseases, the results we found in these two ODCs may not be applicable for all the characteristics of the two disease types. Moreover, there is inevitably some overlap among the communities. Patients with physical illness may also suffer from mental distress. Therefore, comparing the differences between patients with psychological and physiological diseases requires more ODC studies and more rigorous user filtering mechanisms.

The second limitation is the methods we used for topic mining and sentiment analysis. Manual coding was used to label the most related topic in each LDA category. This process could cause deviations in theme classification to some extent. A more reasonable topic model or clustering method can be considered in the future. TextMind was used for sentiment analysis, which includes multiple dimensions to describe the users’ mental states. We compared the two dimensions in it (positive emotion and negative emotion) to identify sentiment polarity, and its effectiveness needs to be further verified. Future work could further investigate this by using a mixed method of machine learning and manual labeling.

Finally, our study did not examine the duration of emotional contagion within the community and the extent to which users were affected. Although previous studies suggested that emotional contagion is prevalent in social communities, we cannot conclude that positive or negative emotion directly caused an impact on the users. We presented the emotional contagion pattern through a weighted social network, and conducting longitudinal studies to analyze the patients’ emotion changes could deepen our understanding of emotional contagion in future work.

## 5. Conclusions

The main purpose of this study was to find the similarities and differences between online psychological and physiological disease communities. Data were collected from Baidu Tieba, which is the largest online forum in China. Topic modeling, sentiment analysis, and social network analysis can effectively capture the characteristics of patients with different diseases. Patients with depression had a great demand for emotional catharsis and preferred to express their emotions at night. They focused more on themselves and did not care about the treatment options. Diabetics advocated maintaining fitness through self-management. Furthermore, emotional contagion patterns were generally different in two communities. In the depression community, there were two obvious interveners spreading positive emotions and more core users in the negative emotional contagion network. In the diabetes community, more users were involved in the positive emotional contagion network and core users in positive and negative emotional contagion networks were basically the same. In summary, our overall finding extends the existing emotional contagion theory to the OHCs by identifying the posts’ sentiment and core users in active group communications. We also contribute practical suggestions for designing ODCs to improve the mental and physical benefits of members. Different and timely interventions should be adopted according to the content and emotional tone of discussions in the whole communities, thus creating a positive atmosphere and promoting the healthy and sustainable development of the communities. The results are also helpful for health experts and practitioners to understand the users’ different informational and emotional needs expressed online and help them better manage their health.

## Figures and Tables

**Table 1:** Data extracted from two communities.

Thread
Thread_id (the unique identifier for a thread)
Author
Portrait (the unique identifier for the author)
Reply_num (number of reply posts)
Title (title of initial post)
Post
Post_id (the unique identifier for a post)
Floor (the floor in its thread, which represents the order of posts, e.g., P_1_ is the first floor)
Author
Portrait (the unique identifier for the author)
Content
Time
Comment_num (number of comments under the post)
Thread_id (the id of a thread that the post belongs to)
Comment
Comment_id (the unique identifier for a comment)
Author
Portrait (the unique identifier for the author)
Content
Time
Post_id (the id of a post that the comment replies to)

**Table 2:** Descriptive statistics of the whole dataset and sample dataset.

Dataset		Depression	Diabetes
Whole dataset	Thread creators	6965	4147
	Threads	12,040	8091
	Average number of threads per creator	1.729	1.951
	Reply posts	140,247	65,688
	Average number of reply posts per thread	11.648	8.119
	Comments	164,100	76,218
Sample dataset	Thread creators	465	319
	Threads	4033	3326
	Average number of threads per creator	8.673	10.426
	Reply posts	25,095	15,508
	Average number of reply posts per thread	6.222	4.663
	Comments	29,175	19,599
	Users involved	7100	3233

**Table 3:** The two-layer theme classification scheme for the texts in online disease communities.

Main Topics	Subtopics	Description
Treatment [16,47]	Drug therapy [19]	Treatment that involves using medications to treat diseases or conditions, usually on a consistent basis.
	Nondrug therapy [19]	Treatments of diseases, which include the hospitalization, psychotherapy and surgery, etc.
Symptoms [48]	Psychological	Abnormal feelings and thoughts of patients due to diseases.
	Physical	Abnormal physical conditions of patients due to diseases.
Self-management [16]	Lifestyle [49,50]	Life record about work, diet, mood or other issues.
	Interventions [51]	The intervention of diseases with food, exercise or other methods to keep healthy or help for recovery in daily life.
Social environment [47]	Relationships [52]	Patients’ relationships with parents, husband or wife, friends, etc.
	Social events	Discussion about social events related to diseases.

**Table 4:** Descriptive statistics for the positive and negative emotional contagion networks.

	Positive Emotional Contagion Network		Negative Emotional Contagion Network	
	Depression	Diabetes	Depression	Diabetes
Nodes	3834	3157	2906	1410
Ties	6757	2910	4358	2481
Average Degree	1.762	1.815	1.500	1.760
Average Clustering Coefficient	0.014	0.027	0.010	0.029
Average Path Length	3.872	3.550	4.078	3.388

**Table 5:** Top 10 participants in subnetworks sorted by out degree.

		Positive Emotional Contagion Network	Negative Emotional Contagion Network
Community	Rank	Participant	Participant
Depression	1	Dep_user_01	Dep_user_11
	2	Dep_user_02	Dep_user_12
	3	Dep_user_03	Dep_user_13
	4	Dep_user_04	Dep_user_03
	5	Dep_user_05	Dep_user_14
	6	Dep_user_06	Dep_user_15
	7	Dep_user_07	Dep_user_16
	8	Dep_user_08	Dep_user_17
	9	Dep_user_09	Dep_user_05
	10	Dep_user_10	Dep_user_18
Diabetes	1	Dia_user_01	Dia_user_01
	2	Dia_user_02	Dia_user_02
	3	Dia_user_03	Dia_user_08
	4	Dia_user_04	Dia_user_03
	5	Dia_user_05	Dia_user_05
	6	Dia_user_06	Dia_user_06
	7	Dia_user_07	Dia_user_10
	8	Dia_user_08	Dia_user_11
	9	Dia_user_09	Dia_user_12
	10	Dia_user_10	Dia_user_13

## Data Availability

Data are available on request from the authors.

## Appendix A

**Table A1:** High probability words in each LDA category.

Topic Label	Category Name	High Probability Words
Topic 0	Psychological	when, feel, myself, state, anxious, now, emotion, breath, practice, recently, start, symptoms, situation, suddenly, easy, before, already, more and more, find, life, work, normal, method, every day, nervous, reaction
Topic 1	Lifestyle	blood glucose, fasting, hour, control, exercise, meal, breakfast, morning, 2hPG, dinner, lunch, around, normal, diet, take medicine, rice, egg, saccharifying, noon, evening, steamed bun, milk, glucometers, weight, 1hPG
Topic 2	Relationships	world, we, leave, may, why, woman, last, time, believe, wife, mental illness, exist, accept, come back, love, feel, see, forever, already, hope, tell, choose, understand, find
Topic 3	Lifestyle	today, a little, feeling, a few days, know, morning, results, tomorrow, yesterday, has been, own, work, already, hospital, every day, go home, eat, hour, sleep, get up, feel bad, afternoon, come back, all day
Topic 4	Drug therapy	health, product, sugar free, food, Chinese medicine, standards, actually, all, become, problem, test, knowledge, institution, sucrose, brands, same, research, body, know, hope, China
Topic 5	Nondrug therapy	they, friends, myself, together, know, hospital, doctor, be hospitalized, feel, communication, family, tell, online, work hard, discharged, hope, chat, encourage, therapy, suggestion, advice, experience
Topic 6	Drug therapy	traditional Chinese medicine, China, Western medicine, medical, science, therapy, develop, professor, TCM, medication, virus, combine, effect, put forward, medical treatment, glucose, theory, verify
Topic 7	Physical	patient, appear, symptom, result in, complication, hypertension, occur, lesion, hyperglycemia, disease, blood vessel, decline, weight, serious, infection, morbidity, long term, abnormal, performance, skin, harm, metabolize
Topic 8	Relationships	ourselves, we, the other people, emotions, they, problem, life, if, psychology, may, heart, a lot of, matter, oneself, need, others, time, behavior, pain, each other, like, certain, thinking, ability, method, different
Topic 9	Self-management	effect, peas, physique, dedicated, ginseng, function, strengthen, constipation, the human body, spleen and stomach, immune, diseases, symptoms, treatment, cold, summer, sun, improve, massage, fungus, efficacy
Topic 10	Nondrug therapy	doctors, hospitals, inspection, diagnosis, food, as a result, be hospitalized, glucose meter, detection, control, bubble, cause, blood sugar, uric acid, starch, children, take medicine, died, data, help, recommend, avoid
Topic 11	Physical	hypoglycemia, secretion, resistance, cell, glucose, rise, injection, reduce, increasing, function, absorption, endocrine, metabolic, blood sugar levels, complications, phase, circumstances, hyperglycemia, change
Topic 12	Social events	highly-skilled doctor, traitor (to China), fraud, these, patient, master, liar, all day, know, so called, Chinese medicine, why, really, magic medicine, doctors, idiot, tell, country, should, bullshit
Topic 13	Physical	feeling, today, uncomfortable, interactive, happy, sad, a little, every day, in the heart, have a headache, sleep, nausea, problem, tomorrow, dizziness, end, recently, have a meal, recurrence, pain, spirit, head, wake up
Topic 14	Relationships	why, like, think, know, others, myself, really, they, don’t want to, hate, fear, parents, always, mom, get married, dare not, special, in fact, as if, now, home, family, children
Topic 15	Psychological	myself, know, really, feeling, pain, now, think, live, don’t want to, every day, hope, life, others, bad, happy, friends, start, emotions, only that, everything, one day, speak, hang on
Topic 16	Self-management	food, edible, diet, contain, control, fat, vitamins, protein, calories, fruit, candy, intake, nutrition, function, vegetables, energy, cholesterol, influence, rise, dietary fiber, diabetic, reduce, improve, carbohydrates
Topic 17	Self-management	exercise, watch out, choice, easy, time, hypoglycemia, avoid, control, skin, suggestion, minutes, the best, diet, unfavorable, fit, keep, strength, summer, appropriate, comparison, activities, autumn, the old
Topic 18	Social events	liar, we, as long as, problem, no matter, function, tell, heart, liver, blood sugar, doctors of traditional Chinese medicine, body, continue, if, complaints, normal, know, propaganda, organs, fool, reversed
Topic 19	Physical	blood sugar, balance, pregnant women, babies, polyuria, western medicine, hyperglycemia, patients, body, resistance, chronic disease, pregnancy, glucose, complications, etiology, symptoms, insulin, rise, absorption
Topic 20	Social events	tong chengkang(a medicine brand), cheats, NMN, sugar, EVIPROSTAT(a medicine brand), cancer, disclose, opposite, post, cure, material, a bottle, fool, repair, efficacy, illness, treatment
Topic 21	Drug therapy	treatment, patients, drugs, insulin, research, control, through, use, function, effect, method, complications, improve, regulate, techniques, clinical, injection, patients, illness, adjustment, dosage, drug use, reduce
Topic 22	Social events	liar, everybody, friend, cure, Chinese medicine, come out, fake medicine, face, patient, post, cure, miracle doctor, advertising, illegal, dummy account, swindling, any, unmasking evidence, hospital,
Topic 23	Relationships	you, energy, children, take medicine, parents, boring, insomnia, family, school, psychological, serious, teachers, question, state, control, anxiety, sleep, drop out of school, cause, teenagers, students, suicide, brain

For confidentiality reasons, the users’ portraits were not displayed in its entirety, and parts of them were replaced with *.

**Table A2:** Top 10 core uses sorted by out degree in four emotional contagion subnetworks.

Community	Subnetworks	User Portrait	Weight	In Degree	Out Degree	Degree	Closeness Centrality	Betweenness Centrality
Depression	Positive Emotional Contagion Network	tb.1.12244748. ******tXNg	2202	0	233	233	0.36	0.00
		tb.1.1e23b567. ******bz3Q	449	10	199	209	0.40	15,532.47
		tb.1.558c46e3. ******crtA	401	63	152	215	0.32	57,836.79
		tb.1.6dd7b726. ******bWag	205	39	86	125	0.47	71,320.66
		tb.1.872d11dd. ******aqwQ	253	80	81	161	0.37	58,913.49
		tb.1.772fca34. ******bO0w	206	42	68	110	0.29	49,018.11
		tb.1.6b52a8de. ******FRfg	180	33	56	89	0.36	33,454.10
		tb.1.145baad2. ******OjqQ	246	1	53	54	0.35	847.83
		tb.1.6a868fff. ******p7JA	150	27	50	77	0.39	41,098.51
		tb.1.727253e4. ******iDHQ	98	29	49	78	0.38	42,429.42
	Negative Emotional Contagion Network	tb.1.f8a40f87. ******mirzA	127	1	68	69	0.30	558.00
		tb.1.f91d2dcc. ******kklg	116	0	63	63	0.29	0.00
		tb.1.e1f676d6. ******L7Hw	92	5	49	54	0.34	3467.80
		tb.1.558c46e3. ******TcrtA	330	133	48	181	0.79	22,191.20
		tb.1.d4ae140. ******ZdHQ	149	7	48	55	0.31	5025.06
		tb.1.ca071b32. ******-x7A	69	2	43	45	0.32	3035.44
		tb.1.fe64a179. ******tJTA	111	0	42	42	0.31	0.00
		tb.1.fc2a69fc. ******80tng	60	0	38	38	0.27	0.00
		tb.1.872d11dd. ******aqwQ	120	46	35	81	0.36	10,620.54
		tb.1.d8d9cbdc. ******g2PQ	69	5	33	38	0.33	4835.55
Diabetes	Positive Emotional Contagion Network	tb.1.6d1455de. ******K4FA	546	83	64	147	0.45	35,034.85
		tb.1.f2c87e61. ******j_GA	663	6	57	63	0.38	2880.09
		tb.1.f1dcd94c. ******8Pdg	190	5	56	61	0.40	2281.25
		tb.1.c895e33f. ******MeWQ	140	15	46	61	0.38	4508.27
		tb.1.fa657784. ******5NkA	320	2	45	47	0.36	590.20
		tb.1.f7cd4ddd. ******bIJQ	68	0	45	45	0.37	0.00
		tb.1.f2758f54. ******hRUg	86	4	38	42	0.36	911.02
		tb.1.efb739dc. ******7v4A	72	7	37	44	0.38	1989.93
		tb.1.a3c72885. ******NjLg	80	18	36	54	0.39	8896.04
		tb.1.ff3e1841. ******6Eow	123	0	29	29	0.35	0.00
	Negative Emotional Contagion Network	tb.1.6d1455de. ******K4FA	1694	81	67	148	0.47	25,521.69
		tb.1.f2c87e61. ******j_GA	765	4	56	60	0.41	863.04
		tb.1.efb739dc. ******7v4A	67	4	43	47	0.37	1753.69
		tb.1.f1dcd94c. ******8Pdg	330	6	42	48	0.40	933.65
		tb.1.fa657784. ******5NkA	618	1	37	38	0.35	142.27
		tb.1.f7cd4ddd. ******bIJQ	55	1	29	30	0.32	307.16
		tb.1.ff3e1841. ******6Eow	99	0	26	26	0.33	0.00
		tb.1.bf01b496. ******oyBg	510	7	25	32	0.39	1107.25
		tb.1.f1b7b55c. ******VPhw	267	6	25	31	0.36	631.77
		tb.1.73ea5320. ******hx5w	122	35	25	60	0.43	6549.71

## References

[1] World Health Organization (2021). Chronic Diseases and Health Promotion. https://www.who.int/chp/about/integrated_cd/en/.

[2] Ruthven I., Buchanan S., Jardine C. (2018). Relationships, environment, health and development: The information needs expressed online by young first-time mothers. J. Assoc. Inf. Sci. Technol..

[3] Zhang S., O’Carol Bantum E., Owen J., Bakken S., Elhadad N. (2017). Online cancer communities as informatics intervention for social support: Conceptualization, characterization, and impact. J. Am. Med. Inform. Assoc..

[4] Della Rosa S., Sen F. (2019). Health topics on Facebook groups: Content analysis of posts in multiple sclerosis communities. Interact. J. Med. Res..

[5] Kaur S., Kaul P., Zadeh P.M. (2020). Monitoring the dynamics of emotions during COVID-19 using Twitter data. Procedia Comput. Sci..

[6] da Silva L.F.C., Barbosa M.W., Gomes R.R. (2019). Measuring participation in distance education online discussion forums using social network analysis. J. Assoc. Inf. Sci. Technol..

[7] Park A., Conway M., Chen A.T. (2018). Examining thematic similarity, difference, and membership in three online mental health communities from Reddit: A text mining and visualization approach. Comput. Hum. Behav..

[8] Tang J., Yu G., Yao X. (2020). A comparative study of online depression communities in china. Int. J. Environ. Res. Public Health.

[9] Oh S. (2012). The characteristics and motivations of health answerers for sharing information, knowledge, and experiences in online environments. J. Am. Soc. Inf. Sci. Technol..

[10] Zhao D., Zhang Q., Ma F. (2020). Communication that changes lives: An exploratory research on a chinese online hypertension community. Libr. Hi Tech.

[11] Naveh S., Bronstein J. (2019). Sense making in complex health situations: Virtual health communities as sources of information and emotional support. Aslib J. Inf. Manag..

[12] Haimson O.L. (2019). Mapping gender transition sentiment patterns via social media data: Toward decreasing transgender mental health disparities. J. Am. Med. Inform. Assoc..

[13] Meng F., Zhang X., Liu L., Ren C. (2021). Converting readers to patients? From free to paid knowledge-sharing in online health communities. Inf. Processing Manag..

[14] Ma D., Zuo M., Liu L. (2021). The information needs of chinese family members of cancer patients in the online health community: What and why?. Inf. Processing Manag..

[15] Lu Q., Song B., Chen J., Xie I., Shen Y. (2021). Information needs and services for autism in China: Is there any gap between them?. Aslib J. Inf. Manag..

[16] Chen A.T. (2012). Exploring online support spaces: Using cluster analysis to examine breast cancer, diabetes and fibromyalgia support groups. Patient Educ. Couns..

[17] Low D.M., Rumker L., Talkar T., Torous J., Cecchi G., Ghosh S.S. (2020). Natural language processing reveals vulnerable mental health support groups and heightened health anxiety on Reddit during COVID-19: Observational study. J. Med. Internet Res..

[18] Gkotsis G., Oellrich A., Hubbard T., Dobson R., Liakata M., Velupillai S., Dutta R. The Language of Mental Health Problems in Social Media. Proceedings of the 3rd Workshop on Computational Linguistics and Clinical Psychology: From Linguistic Signal to Clinical Reality.

[19] Liu J., Kong J., Zhang X. (2020). Study on differences between patients with physiological and psychological diseases in online health communities: Topic analysis and sentiment analysis. Int. J. Environ. Res. Public Health.

[20] Pennebaker J.W., Mehl M.R., Niederhoffer K.G. (2003). Psychological aspects of natural language use: Our words, our selves. Annu. Rev. Psychol..

[21] Zhao K., Greer G., Yen J., Mitra P., Portier K. (2015). Leader identification in an online health community for cancer survivors: A social network-based classification approach. Inf. Syst. e-Bus. Manag..

[22] Zhao K., Yen J., Greer G., Qiu B., Mitra P., Portier K. (2014). Finding influential users of online health communities: A new metric based on sentiment influence. J. Am. Med. Inform. Assoc..

[23] Yang J., Zou X., Zhang W., Han H. (2021). Microblog sentiment analysis via embedding social contexts into an attentive LSTM. Eng. Appl. Artif. Intell..

[24] Zou X., Yang J., Zhang J. (2018). Microblog sentiment analysis using social and topic context. PLoS ONE.

[25] Kramer A.D.I., Guillory J.E., Hancock J.T. (2014). Experimental evidence of massive-scale emotional contagion through social networks. Proc. Natl. Acad. Sci. USA.

[26] Hill A.L., Rand D.G., Nowak M.A., Christakis N.A. (2010). Emotions as infectious diseases in a large social network: The SISa model. Proc. R. Soc. B Biol. Sci..

[27] Barsade S.G. (2002). The Ripple effect: Emotional contagion and its influence on group behavior. Adm. Sci. Q..

[28] Yoo W., Namkoong K., Choi M., Shah D.V., Tsang S., Hong Y., Aguilar M., Gustafson D.H. (2014). Giving and receiving emotional support online: Communication competence as a moderator of psychosocial benefits for women with breast cancer. Comput. Hum. Behav..

[29] Kim E., Han J.Y., Shah D., Shaw B., McTavish F., Gustafson D.H., Fan D. (2011). Predictors of supportive message expression and reception in an interactive cancer communication system. J. Health Commun..

[30] Namkoong K., McLaughlin B., Yoo W., Hull S.J., Shah D.V., Kim S.C., Moon T., Johnson C., Hawkins R., McTavish F.M. (2013). The effects of expression: How providing emotional support online improves cancer patients’ coping strategies. J. Natl. Cancer Inst. Monogr..

[31] Uchino B.N. (2004). Social Support and Physical Health: Understanding the Health Consequences of Relationships.

[32] Albrecht T.L., Goldsmith D.J., Thomson T., Dorsey A.M., Miller K.I., Parrott R. (2003). Social Support, Social Networks, and Health. Handbook of Health Communication.

[33] Wikipedia (2019). Baidu Tieba. https://en.wikipedia.org/wiki/Baidu_Tieba.

[34] Liu C., Lu X. (2018). Analyzing hidden populations online: Topic, emotion, and social network of HIV-related users in the largest chinese online community. BMC Med. Inform. Decis. Mak..

[35] Park A., Conway M. (2017). Longitudinal changes in psychological states in online health community members: Understanding the long-term effects of participating in an online depression community. J. Med. Internet Res..

[36] Park A., Hartzler A.L., Huh J., McDonald D.W., Pratt W. (2015). Homophily of vocabulary usage: Beneficial effects of vocabulary similarity on online health communities participation. AMIA Annu. Symp. Proc..

[37] Blei D.M., Ng A.Y., Jordan M.I. (2003). Latent Dirichlet allocation. J. Mach. Learn. Res..

[38] Wang X., Parameswaran S., Bagul D.M., Kishore R. (2018). Can online social support be detrimental in stigmatized chronic diseases? A quadratic model of the effects of informational and emotional support on self-care behavior of HIV patients. J. Am. Med. Inform. Assoc..

[39] Gao R., Hao B., Li H., Gao Y., Zhu T., Immamura K., Usui S., Shirao T., Kasamatsu T., Schwabe L., Zhong N. (2013). Developing Simplified Chinese Psychological Linguistic Analysis Dictionary for Microblog. Brain and Health Informatics, Proceedings of the International Conference on Brain and Health Informatics Maebashi, Japan, 29–31 October 2013.

[40] Bellon-Harn M.L., Ni J., Manchaiah V. (2020). Twitter usage about autism spectrum disorder. Autism.

[41] Wang Y.F., Zhao Y.P., Zhang J.Q., Bian J., Zhang R. (2020). Detecting associations between dietary supplement intake and sentiments within mental disorder tweets. Health Inform. J..

[42] Zhao Y.H., Da J.W., Yan J.Q. (2021). Detecting health misinformation in online health communities: Incorporating behavioral features into machine learning based approaches. Inf. Process. Manag..

[43] Cobb N.K., Graham A.L., Abrams D.B. (2010). Social network structure of a large online community for smoking cessation. Am. J. Public Health.

[44] Parameswaran S., Kishore R. Social Support in Online Health Communities: A Social-Network Approach. Proceedings of the 2018 ACM SIGMIS Conference on Computers and People Research.

[45] Yang F., Zhong B., Kumar A., Chow S.-M., Ouyang A. (2017). Exchanging social support online: A longitudinal social network analysis of irritable Bowel Syndrome patients’ interactions on a health forum. J. Mass Commun. Q..

[46] Landis J.R., Koch G.G. (1977). The measurement of observer agreement for categorical data. Biometrics.

[47] Feldhege J., Moessner M., Bauer S. (2020). Who says what? Content and participation characteristics in an online depression community. J. Affect. Disord..

[48] Zhao Y., Chen B., Zhang J., Ding Y., Mao J., Zhou L. (2018). An investigation on the evolution of diabetes data in social Q&A logs. Data Inf. Manag..

[49] Bi Q., Shen L., Evans R., Zhang Z., Wang S., Dai W., Liu C. (2020). Determining the topic evolution and sentiment polarity for albinism in a chinese online health community: Machine learning and social network analysis. JMIR Med. Inform..

[50] Ji Z.C., Zhang Y.Y., Xu J., Chen X.L., Wu Y.H., Xu H. (2017). Comparing cancer information needs for consumers in the us and china. Medinfo 2017 Precis. Healthc. Through Inform..

[51] Cronin R.M., Fabbri D., Denny J.C., Jackson G.P. (2015). Automated classification of consumer health information needs in patient portal messages. AMIA Annu. Symp. Proc..

[52] Zhao Y., Zhang J., Wu M. (2019). Finding users’ voice on social media: An investigation of online support groups for autism-affected users on Facebook. Int. J. Environ. Res. Public Health.

[53] Blondel V.D., Guillaume J.-L., Lambiotte R., Lefebvre E. (2008). Fast unfolding of communities in large networks. J. Stat. Mech. Theory Exp..

[54] Lancichinetti A., Fortunato S. (2009). Community detection algorithms: A comparative analysis. Phys. Rev. E.

[55] Shah B.B., Nieweglowski K., Corrigan P.W. (2020). Perceptions of difference and disdain on the self-stigma of mental illness. J. Ment. Health.

[56] Corrigan P.W., Watson A.C. (2002). Understanding the impact of stigma on people with mental illness. World Psychiatry.

[57] Latalova K., Kamaradova D., Prasko J. (2014). Perspectives on perceived stigma and self-stigma in adult male patients with depression. Neuropsychiatr. Dis. Treat..

[58] Arnaez J.M., Krendl A.C., McCormick B.P., Chen Z., Chomistek A.K. (2020). The association of depression stigma with barriers to seeking mental health care: A cross-sectional analysis. J. Ment. Health.

[59] Halter M.J. (2004). The stigma of seeking care and depression. Arch. Psychiatr. Nurs..

